# Neutrophil activation identifies patients with active polyarticular gout

**DOI:** 10.1186/s13075-020-02244-6

**Published:** 2020-06-18

**Authors:** D. Vedder, M. Gerritsen, B. Duvvuri, R. F. van Vollenhoven, M. T. Nurmohamed, C. Lood

**Affiliations:** 1 Amsterdam Rheumatology & Immunology Center, Reade, 1056 AB Amsterdam, Netherlands; 2 Amsterdam Cardiovascular Sciences, Amsterdam, Netherlands; 3 Division of Rheumatology, University of Washington, Seattle, WA USA; 4 Amsterdam University Medical Center, Amsterdam, Netherlands

**Keywords:** Gout, Crystal-induced arthritis, Neutrophils, Innate immunity, Disease activity

## Abstract

**Background:**

Gout is the most prevalent inflammatory arthritis in developed countries. A gout flare is mediated by phagocytosis of monosodium urate crystals by macrophages and neutrophils leading to subsequent activation of neutrophils contributing to synovitis, local joint destruction, and systemic inflammation. We hypothesize that biomarkers from activated neutrophils reflect gout disease activity.

The objective of this study therefore was to investigate the clinical utility of neutrophil-derived biomarkers in gout disease activity.

**Methods:**

Plasma samples from 75 gout patients participating in the “Reade gout cohort Amsterdam” were compared with 30 healthy controls (HC). Levels of neutrophil extracellular traps (NETs) and neutrophil activation markers (calprotectin and peroxidase activity) were analyzed by ELISA and fluorimetry, compared to healthy controls, and related to markers of inflammation and disease activity.

**Results:**

Levels of NETs, as well as neutrophil activation markers, were increased in gout patients compared to HC (*p* < 0.01). No associations were found between markers of cell death (cell-free DNA and NETs) and disease activity. Cell-free levels of genomic DNA were elevated among gout patients compared to HC (*p* < 0.05) and related to the number of gout attacks in the last year (*β* = 0.35, *p* < 0.01). Peroxidase activity correlated with disease activity (RAPID score: *β* = 0.49, *p* < 0.01, MHAQ: *β* = 0.66, *p* < 0.01) and inflammation markers (CRP: *β* = 0.25, *p* = 0.04, and ESR: *β* = 0.57, *p* < 0.001). Involvement of ankle or wrist resulted in significant higher peroxidase levels compared to mono-articular disease (*β* = 0.34, *p* < 0.01), indicating that peroxidase activity is a marker of poly-articular gout. Calprotectin (S100A8/A9) correlated with the inflammation marker CRP (*β* = 0.23, *p* = 0.05) and morning stiffness, especially in patients with chronic poly-articular gout (*β* = 0.71, *p* < 0.01).

**Conclusions:**

Neutrophil activation markers are associated with characteristics of active, polyarticular gout. Furthermore, NETs are present in the peripheral blood of gout patients. However, NETs do not associate with markers of disease activity or inflammation. Future research should point out if peroxidase and calprotectin could be used in clinical practice as biomarkers for monitoring gout disease activity.

## Background

A gout flare is an inflammatory response to deposited monosodium urate (MSU) crystals, mediated by activation of the innate immune system with attraction and activation of macrophages and neutrophils. During phagocytosis of the crystals, macrophages activate the inflammasome with subsequent release of interleukin (IL-) 1B and other pro-inflammatory cytokines [1]. These cytokines increase the attraction and activation of neutrophils, which in turn participate in host defense through several mechanisms, including phagocytosis of the MSU crystals and release of various proteolytic enzymes, as well as formation of neutrophil extracellular traps (NETs) [2]. NET formation is a neutrophil cell death process in which DNA is extruded together with cytoplasmic and granular contents to trap and eliminate extracellular pathogens. Although beneficial from a host-pathogen perspective, excessive neutrophil activation has been linked to inflammation and autoimmunity in various conditions, including rheumatoid arthritis (RA), systemic lupus erythematosus (SLE), and gout [3, 4]. In RA and SLE, NETs are thought to be induced through cytokine- and immune complex-mediated neutrophil activation and their presence has been associated with activation of systemic inflammatory markers [5] and increased risk of systemic organ damage [4, 6]. In in vitro, as well as in animal models of gout, NETs have been found to partake in disease pathogenesis, with NETs being either pro-inflammatory or anti-inflammatory depending on the composition of the NETs [3]. In the confined space of a joint, continuous infiltration of neutrophils and subsequent generation of NETs will induce a phenomenon named aggregated NETs, through which neutrophil-derived proteases degrade inflammatory cytokines and partake in resolution of inflammation [7]. Thus, NETs are thought to partake in both induction and resolution of gout. However, even though local NET formation is suggested to play a crucial role in the gout pathogenesis, as demonstrated in in vitro and in vivo in animal models [8], the role of neutrophil activation and circulating NETs in systemic inflammation in gout patients has not been carefully investigated.

Our objective was therefore to investigate evidence of systemic neutrophil activation in gout patients. In addition, we aimed to study the clinical utility of neutrophil-derived biomarkers in relation to disease activity in gout patients. Given prior in vitro and in vivo data from animal models, we hypothesized that uric acid crystals, either in synovial fluid or deposited in tissue or perhaps even in peripheral blood, will activate neutrophils to release neutrophil-derived activation factors as well as undergoing NET formation. Based on the above-mentioned studies, we anticipate these processes of neutrophil activation and death to be inflammatory, thus contributing to disease activity and systemic inflammation.

## Methods

### Patient cohort

A cross-sectional study was performed. Plasma samples from 75 consecutive gout patients participating in the “Reade gout cohort Amsterdam, the Netherlands” were analyzed and compared with 30 healthy individuals. The Reade gout cohort includes patients with a clinical diagnosis of gout, confirmed by a rheumatologist. All patients fulfilled the EULAR/ACR criteria for the diagnosis of gout [9]. Clinical data were collected on demographics, gout history and disease activity, comorbidities, and medication use. Patient-reported disease activity was measured with the RAPID3 questionnaire, including a Modified Health Assessment Questionnaire (MHAQ), pain and global health assessment (scale 0–10). Patients were asked to fill out this questionnaire within 3 days before or after the study visit [10, 11]. Clinical measurements of disease activity included tender and swollen joint count, measurement of subcutaneous tophi, anthropometry, and lab variables. The study was approved by the regional ethics board (METC Slotervaart and Reade Amsterdam), and informed written consent was obtained from all participants in accordance with the Helsinki Declaration. For the healthy control group, demographics (gender, age) and levels of neutrophil activation markers were obtained.

### Neutrophil activation assays

Blood samples from the gout cohort were collected at the same visit and processed at Reade Amsterdam. For plasma preparation, EDTA blood samples were centrifuged at 1711*g* for 10 min. Levels of NETs were analyzed using an MPO-DNA ELISA, as described previously [12]. In brief, a 96-well microtiter plate (Corning) was coated with anti-MPO antibody (4 μg/mL, Biorad) overnight at 4 °C, followed by blocking with 1% bovine serum albumin (BSA) in PBS for 2 h at room temperature. After blocking, plasma samples (10% in PBS-BSA) were added and incubated overnight at 4 °C. For detection, anti-dsDNA-HRP antibody (diluted 1/100, Roche Diagnostic) was added for 2 h, room temperature. The reaction was developed with 3,3′,5,5′-tetramethylbenzidine (TMB, BD Biosciences) and ended by the addition of 2 N sulfuric acid. Absorbance was measured at 450 nm by a plate reader (Synergy, BioTek).

Peroxidase activity was analyzed as previously described. In brief, plasma samples (10%) were incubated with TMB (3,3′,5,5′-tetramethylbenzidine, BD Biosciences) at a final volume of 100 μL for 30 min at room temperature. The reaction was ended by addition of 2 N sulfuric acid. The absorbance was analyzed by a plate reader at 450 nm. Values are reported as mU/mL using HRP (Sigma) as standard curve. Levels of calprotectin (S100A8/A9) were analyzed in plasma using a commercial ELISA kit according to the manufacturer’s instructions (R&D Systems). Levels of cell-free (cf) DNA, nuclear (n), and mitochondrial (mt) DNA were analyzed by fluorimetry and qPCR, respectively, as described previously [12, 13].

All of the analyzed markers were compared between gout patients and healthy controls. In gout patients, markers of neutrophil activation and NET formation were related to clinical markers of disease activity including disease duration as continuous and dichotomous variable (< 1 year vs > 1 year history of gout flares), amount and duration of gout flares, amount of involved joints (mono- vs poly-articular gout), uric acid levels, and presence of tophi. Poly-articular gout was defined as involvement of different joints (> 1 unique joint) in the past.

### Statistical analysis

Patient characteristics were expressed as number (percentage), means (± standard deviation (SD)), when normally distributed, or median [interquartile range IQR], when skewed distributed. Normally distributed variables were analyzed with linear regression analysis. For sample sets with a non-Gaussian distribution, Mann-Whitney *U* test and Spearman’s correlation test were used, as applicable. The 90th percentile of the healthy controls was used to define positive samples. Results for the gout cohort were adjusted for possible confounding by age and gender. All analyses were considered statistically significant at *p* < 0.05.

## Results

### Patients characteristics

Patient characteristics are presented in Table 1. Patients are predominantly Caucasian male with a mean age of 58 years. The mean gout disease duration was 7 years. The number of self-reported flares since diagnosis differed extensively between patients with a median of 10 flares (IQR 4–22 flares). During the last year, patients experienced between 0 and 10 flares (mean 3.45 ± 2.55). The median duration of a gout flare was 5 days (IQR 3–7) while the last experienced attack was of significantly longer duration (median 12 days, IQR 4–35 days). In 60% of the patients (*n* = 45), MSU crystal confirmation testing was performed and MSU crystals were actually present in 41 out of these 45 patients. Twenty-two patients (29%) had limited joint involvement (mono-articular gout), mostly podagra, while 53 patients (71%) had experienced gout attacks in more than one joint in the past (previously defined as poly-articular gout) with visible tophi in 29% of all patients. The median VAS disease activity was 18.5 mm (IQR 1–48.5). In most cases, colchicine was used during the last gout flare (69%) followed by NSAIDs (55%) and corticosteroids (43%), or a combination of these drugs. Two third of all patients (66%) received urate-lowering therapy at time of baseline, predominantly allopurinol (96%). Mean serum urate level at baseline was 0.42 mmol/l (7 mg/dl).

**Table 1:** Patient characteristics gout cohort

Characteristics	Gout cohort (***N*** = 75)	Control group (***N*** = 30)
Gender (*N*, % male)	68 (89.5%)	14 (46.7%)
Age in years (mean, SD)	58 (± 11.7)	49 (± 15.2)
Disease duration in years (median, IQR)	4 [2–10]	N.A.
Mono-articular gout (*N*, %)	22 (29%)	N.A.
Poly-articular gout (*N*, %)	53 (71%)	N.A.
Subcutaneous tophi (*N*, %)	22 (29%)	N.A.
VAS pain (median, IQR)	11 [1–45]	N.A.
VAS disease activity (median, IQR)	19 [1–48.5]	N.A.
RAPID score total (median, IQR)	4.3 [1.8–6.0]	N.A.
Urate lowering therapy (*N*, %)	49 (66%)	N.A.
Smoking, yes (*N*, %)	13 (17.1%)	5 (17%)
BMI (mean, SD)	30.4 (± 4.9)	24.1 (± 3.2)
Serum urate (mmol/L) (mean, SD)	0.42 (± 0.09)	N.D.
BSE [median, IQR]	7 [4.5–10.5]	N.D.
CRP [median, IQR]	2.2 [1.1–3.7]	N.D.
Creatinine (mean, SD)	98.6 (28.3)	N.D.
ALAT (mean, SD)	37.5 (18.8)	N.D.

*N.A.* not applicable, *N.D.* not determined

### Neutrophil activation markers are elevated in patients with poly-articular gout

**Fig. 1:**
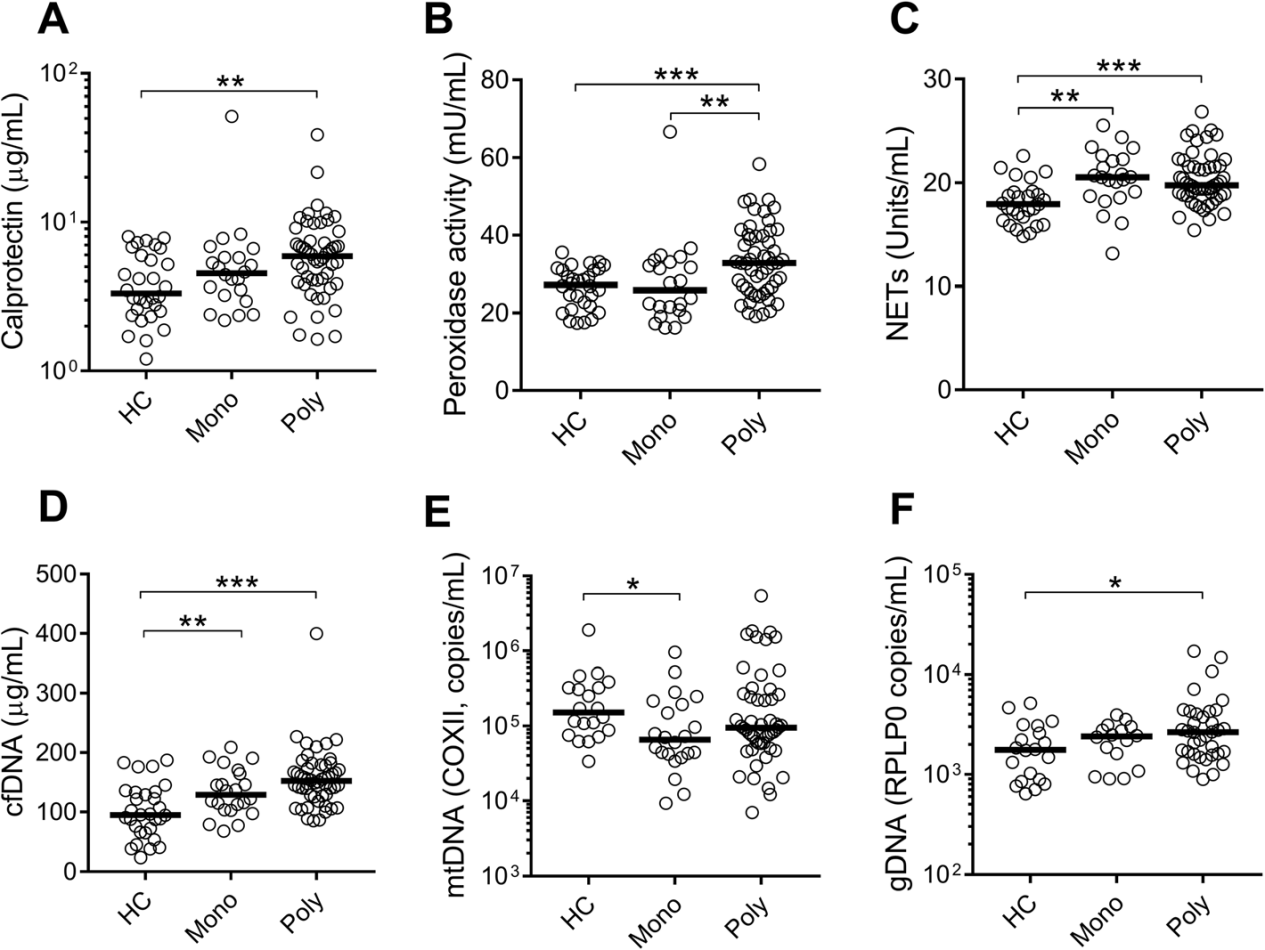
Neutrophil activation in patients with polyarticular gout. Patients were stratified into monoarticular (mono) and polyarticular (poly) gout and assessed for levels of **a** calprotectin, **b** peroxidase activity, **c** NETs, **d** cell-free DNA, **e** mitochondrial DNA, and **f** genomic DNA in plasma. Levels were compared to healthy controls (HC). All statistical analyses were done with Mann-Whitney *U* test with *p < 0.05, **p < 0.01, and ***p < 0.001

We found levels of neutrophil activation markers, calprotectin as well as peroxidase activity, to be elevated in patients with poly-articular gout, but not in mono-articular gout as compared to healthy individuals (*p* < 0.01, Fig. 1a, b). In contrast, markers of neutrophil cell death, including NETs and cfDNA, were elevated in both mono-articular and poly-articular gout as compared to healthy individuals (*p* < 0.01, Fig. 1c, d). Analyzing the source of the extracellular DNA, genomic DNA but not mitochondrial DNA levels were elevated in poly-articular gout (Fig. 1e, f).

### Neutrophil activation markers are associated with disease activity

**Fig. 2:**
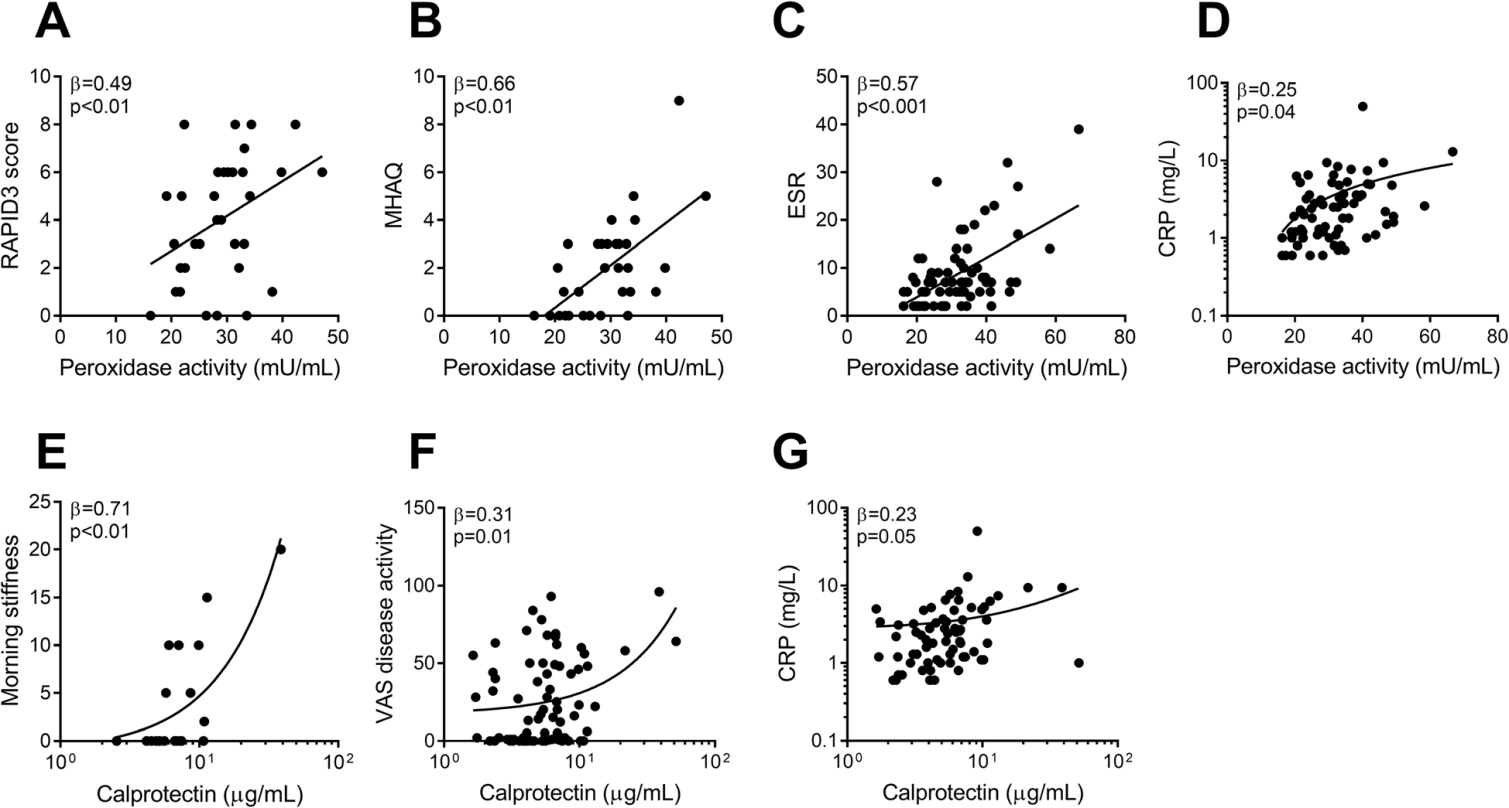
Neutrophil activation markers are associated with disease activity. Levels of peroxidase activity were correlated with markers of disease activity and inflammation including **a** RAPID3 score, **b** MHAQ, **c** erythrocyte sedimentation rate (ESR), and **d** C-reactive protein (CRP). Levels of calprotectin were correlated with markers of disease activity, **e** RAPID morning stiffness, **f** VAS disease activity score, and **g** CRP. All statistical analyses were done with linear regression adjusted for age and gender

No associations were found between markers of cell death (cfDNA and NETs) and disease activity, besides the association between cfDNA and the amount of gout attacks in the last year (*β* = 0.35, *p* < 0.01). However, markers of neutrophil activation (peroxidase and calprotectin) were associated with several indices of gout disease activity. Peroxidase activity correlated with disease activity (RAPID3 score: *β* = 0.49, *p* < 0.01, MHAQ: *β* = 0.66, *p* < 0.01, Fig. 2a, b) and to lesser extent with VAS pain (*β* = 0.25, *p* = 0.04). Furthermore, peroxidase activity correlated well with inflammation markers (CRP: *β* = 0.25, *p* = 0.04 (Fig. 2c), and ESR: *β* = 0.57, *p* < 0.001). Involvement of ankle or wrist resulted in significant higher peroxidase levels (*β* = 0.34, *p* < 0.01), consistent with peroxidase activity being a marker of poly-articular gout (*β* = 0.25, *p* = 0.04). Calprotectin levels correlated with VAS disease activity (*β* = 0.31, *p* = 0.01, Fig. 2f), duration of morning stiffness in patients with poly-articular gout (*β* = 0.71, *p* < 0.01, Fig. 2e), and inflammation marker CRP (*β* = 0.23, *p* = 0.05, Fig. 2g). The appearance of tophi (in 21 patients) was not significantly associated with the level of the neutrophil activation markers (peroxidase: *ß* = 0.07*,* calprotectin*: ß* = 0.19*).* In contrast to neutrophil biomarkers, ESR and CRP were not associated with the numbers of involved joints (CRP: *ß* = 0.10, *p* = 0.38, ESR: *ß* = 0.14, *p* = 0.22) or patient related outcomes as VAS pain (CRP: *ß* = 0.14, *p* = 0.24, ESR: *ß* = 0.16, *p* = 0.19) or VAS disease activity (CRP: *ß* = 0.10, *p* = 0.40, ESR: *ß* = 0.07, *p* = 0.59), but only with disease duration (CRP: *ß* = 0.27, *p* = 0.02, ESR: *ß* = 0.27, *p* = 0.02).

## Discussion

Our main finding is that neutrophil activation markers correlate well with different markers of disease activity, especially with characteristics of active disease and in polyarticular gout. In our opinion, this is an interesting and clinically relevant finding that could well be indicative of (chronic) systemic inflammation in gout. Furthermore, this study, for the first time, demonstrates the presence of NETs in peripheral blood of gout patients. Earlier studies have described neutrophil activation and NET formation locally in tophi and in the precursor form of urate micro aggregates (UMA) but not widespread in the circulation [8].

An explanation to the presence of NETs and neutrophil activation markers in the peripheral blood of gout patients could be the presence of small circulating MSU crystals which activate neutrophils in the circulation leading to phagocytosis and afterwards NET formation [8]. Another explanation could be that local inflammation triggers a systemic inflammatory environment, priming neutrophils for activation and NET formation even in absence of the direct stimulus of a crystal.

NETs are well-known contributors to inflammation, with several damage associated molecule patterns (DAMPs), such as NET-derived DNA, engaging TLRs and other PRR to induce inflammatory responses [12]. Recent work from our group demonstrates that levels of NETs are associated with disease activity and inflammation in two rheumatic diseases: juvenile dermatomyositis and rheumatoid arthritis [13, 14]. Although elevated, the levels of NETs did not correspond with disease activity in our gout cohort. This finding is similar to earlier findings in SLE patients where levels of NETs were elevated though not associated with ongoing disease activity [6]. It is an unexpected and interesting finding, suggesting that what we define as NETs may be a broad definition of more or less pathogenic DNA complexes. Indeed, we, and others, have demonstrated that the cargo of NETs varies depending on mechanism of induction, with certain NETs containing more granular enzymes [15] and other NETs containing more oxidized mitochondrial DNA, the latter being highly inflammatory [12]. Given that the extracellular DNA content (such as NETs) was enriched for genomic DNA, and not inflammatory mitochondrial DNA, this may in part explain the lack of association between NETs and disease activity in these gout patients [13]. Furthermore, pioneering work from the group of Herrmann et al. has suggested that gout-derived NETs, due to their aggregated status, have enhanced proteolytic capacity, acting to suppress inflammation through degradation of inflammatory cytokines. Further studies are needed to investigate the composition of the gout-derived NETs and determine why they are less inflammatory, other than the potential presence of proteases [2].

In clinical practice, specific biomarkers for disease activity in gout are lacking. The level of serum urate is associated with the risk of acute arthritis, and lowering serum urate levels leads to a reduction of tissue deposits and risk of flares [16]. On the other hand, serum urate has been shown to be only weakly associated with patient subjective outcomes such as disability and health-related quality of life (HRQOL) [17]. Our findings indicate that neutrophil activation markers are promising novel biomarkers for both clinical and patient reported outcomes of disease activity in gout. These observations however should be validated in other large gout cohorts. In this cohort, there were differences in baseline characteristics between the two groups (age and gender). Earlier cohort studies however have showed no association between age, gender, and the levels of neutrophil activation markers or NET formation [6]. Similarly, also in the current study, we found no association between levels of neutrophil activation and cell death markers on age and gender. Currently, we are investigating the effect of therapy, especially urate lowering therapy on disease activity as well as neutrophil activation markers. These future results will further clarify the value of these biomarkers in monitoring of disease activity.

## Conclusions

Neutrophil activation markers are associated with characteristics of active polyarticular gout. Furthermore, NETs are present in the peripheral blood of gout patients: however, they do not associate with markers of disease activity or inflammation. Future research should point out if peroxidase and calprotectin could be used in clinical practice as biomarkers for monitoring gout disease activity.

## Data Availability

The datasets used and/or analyzed during the current study are available from the corresponding author on reasonable request.
